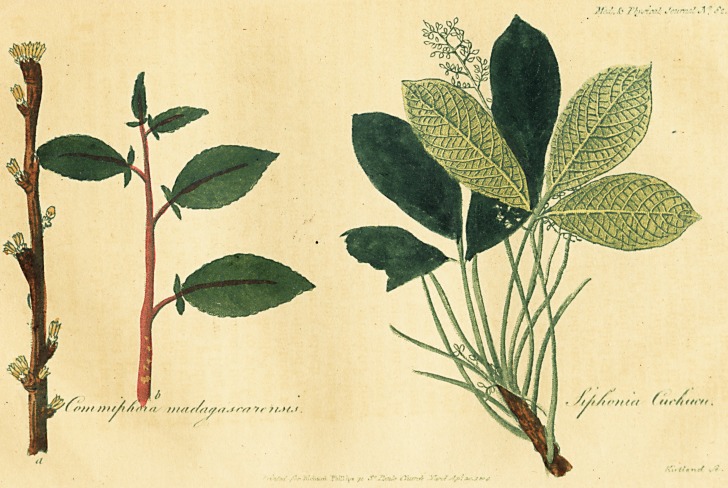# On the Plants from Which the Elastic Resin Is Obtained

**Published:** 1804-05-01

**Authors:** 


### *On the* Plants *from which the* Elastic Resin *is obtained.* *By* Prof. Willdenow.

( With Two coloured Engravings. )

THE remarkable substance known by the name of elastic resin or caoutchouc is imported to us in great quantities from South America in various forms, generally in the shape of bottles, and it is employed in Europe for several medical and chirurgical purposes, for making catheters, trusses, syringes, &c. and also for rubbing out black pencil marks. It was long known to be a vegetable production, and the inspissated juice of a tree, but it was a long time before this was botanically examined. Linnæus thought the elastic gum to be prepared from a tree to which he gave the name of cecropia peltata, and he firmly believed that this was the only tree from which that substance was obtained. It has however since been ascertained by modern botanists, that there are several trees which yield elastic resin, but especially, that the caoutchouc which is imported into Europe, is never taken from cecropia peltata, but from a far different plant. It appears moreover from the researches of modern chemists, that this substance is likewise contained in several European plants, namely, in the berries of viscum album, though it is never to be met with in any European plant in so pure a state as it is observed in several trees of hot climates. The younger Linnæus takes the Jatropha elastica to be the true elastic resin tree, which he learned from Aublet's work (Histoire des Plantes de la Gujane Française, tom. 335, p. 871) who found this tree only with fruit; and Linnæus concluded from the form of the latter, that the elastic gum tree which Aublet calls hevea gujanensis, belonged to the genus Jatropha. Prof. Richard however discovered the same tree during his travels in Gujana, in flower, and at the same time with fruit, and from his accurate examination of both, it appeared to make a new genus which is now called Siphonia. Mr. Richard found but one species which bears the systematic name of Siphonia cahuchu, of which a branch is represented in Fig. 1, on a smaller scale, from a specimen in my collection. Siphonia cahuchu, (Siphonia Elastica, Woodville, Medical Botany, tom. 4) belongs to the Linnæan class of Monœnia, Monadelphia. It is a large tree, growing to the height of sixty feet and more; the bark of the branches is smooth and grey-brown. Leaves on b

*Commiphora madagascarensis.*

a

*Siphonia Cuchucu.*

*Kirtland Sc.*

*Printed for Richard Phillips 72 St. Pauls Church Yard Apl. 20.1804*

on long foot-stalks, and very close together. Leaflets half a foot long, elliptical, pointed, entire, coriaceous, on the upper side smooth and on the under side whitish, with very short, fine, and close hairs. From the axilla of the leaves arises a panicula of small white flowers, longer than the leaves. Male flowers have a monopetalous, campanulate calyx, which is five-cleft. Corolla none. Filaments in a column with five ovate antheræ. Female flowers cleft in teeth which are recurved and deciduous; germen roundish and a little conoid, style none, stigmata three, emarginated, depressed, capsule woody, three-parted, three-celled, in each cell one seed of the size of a sweet almond, elliptical, spotted, and covered with a thin and brittle epidermis. The seeds are said to be eagerly sought for by the Americans and to be a very palatable food. The manner of obtaining elastic resin from this tree is by making incisions in the trunk of the tree, from which a milky juice issues which is received into earthen vessels. On exposure to the air this juice gradually inspissates into the known elastic resin. In America it is employed for different purposes, and they make of it bottles, boots, flambeaux, &c.

Besides this tree, which is now pretty well ascertained, another has been discovered in the Isle of Madagascar, yielding a substance which perfectly resembles that of the Siphona, and said to be obtained by similar proceedings; and it is to be expected, that several other lactescent trees of warm climates may produce such a substance. This tree is called by Mr. Jacquin, Commiphora Madagascarensis. On tab. 2, *(a)* a branch with flowers is represented in natural magnitude; and in *(b)* a branch with leaves lessened by half. It belongs to the class of Diœcia, and the male flowers are only as yet known. Mr. Jacquin gives of it the following description.—The branches are round, covered with a yellowish bark which adheres loosely, leaves oblong, blunt, finely serrated, having at their base two small foliaceous appendices. Flowers yellowish, small, appearing before the eruption of the leaves; they are deciduous. Calyx bell shaped, cleft into four short teeth. Corolla, consisting of four lanceolate petals which surround eight subuliform filaments, four of which are shorter than the rest. Antheræ erect and oblong. Pistillum and fruit unknown.

## Figures and Tables

**Figure f1:**